# Weight trajectories and obesity remission among school-aged children

**DOI:** 10.1371/journal.pone.0290565

**Published:** 2023-09-20

**Authors:** Byron A. Foster, Emile Latour, Jeong Youn Lim, Kelsey Weinstein

**Affiliations:** 1 Department of Pediatrics, Oregon Health & Science University, Portland, Oregon, United States of America; 2 School of Public Health, Oregon Health & Science University and Portland State University, Portland, Oregon, United States of America; 3 Biostatistics Shared Resource, Knight Cancer Institute, Oregon Health & Science University, Portland, Oregon, United States of America; Emory University, UNITED STATES

## Abstract

**Background:**

Many studies examining weight trajectories have used adiposity measures shown to be problematic for trajectory analysis in children with obesity, and remission of obesity remains poorly understood.

**Objectives:**

To describe weight trajectories for school-aged children, the rate of obesity remission and factors associated.

**Methods:**

Children between 6 and 11 years of age with ≥3 valid height and weight measurements from an Oregon hospital-system over a minimum six-month period were included. Percent distance from the median body mass index (BMI) was used for modeling. Latent class analysis and linear mixed models were used to classify children based on their weight trajectory.

**Results:**

We included 11,247 subjects with a median of 2.1 years of follow-up, with 1,614 (14.4%) classified as overweight and 1,794 (16.0%) classified as obese. Of subjects with obesity, 1% experienced remission during follow-up, whereas 23% of those with overweight moved to within a healthy weight range. Latent class analysis identified three classes within each weight-based stratum over time. The majority of children with overweight or obesity had a flat trajectory over time. Lower socioeconomic status was associated with a worsening trajectory. Latent class models using alternate measures (BMI, BMI z-scores, tri-ponderal mass index (TMI)) differed substantially from each other.

**Conclusions:**

Obesity remission was uncommon using the adiposity metric of distance from the median though transition from overweight to healthy weight was more common. Children with low socioeconomic status have worse trajectories overall. The choice of adiposity metric may have a substantial effect on the outcomes.

## Introduction

Remission of obesity in children has been under-studied. Much of the research focus for childhood obesity has been on risk factors for having or developing obesity, and the factors associated with remission may not be the same. A better understanding of factors associated with remission can inform programs to facilitate remission. There are recent trajectory analyses that provide some information on the rate of obesity remission and associated factors. Luan et al. used a nationally-representative sample of 5–14 year old children in the United States and identified 10% of children experienced obesity remission [1]. Remission varied substantially by socioeconomic status with those in the highest category having twice the remission rate as those in the lowest category [1]. Chen et al. reported on a cohort of Texas children followed from age five to age ten and found five different groups of trajectories—two trajectories composed most of the sample—stable healthy weight (51%) and stable overweight or obese (22%) [2]. A minority of children (8%) went from an overweight or obese weight in kindergarten towards a healthy weight by 5^th^ grade [2], and socioeconomic status (SES) at the school level and individual race or ethnicity were not associated with obesity remission. A recent study from China looking at children 6–11 years old using BMI modeling showed that 12% of children went from obesity to a healthy weight and an additional 6% went from obesity to overweight [3]. Other cohorts have identified groups of children with decreasing trajectories ranging from 6–20%—these studies used different modeling to examine trajectories, not remission or changing categories per se [4–6]. The variation in the observed proportion with decreasing trajectories is likely partially driven by differences in methodology of modeling and partially by sample variation.

Other cohort studies have identified multiple latent classes of weight trajectories with no classes or groups of children with obesity remission [7, 8]. In one example of children followed annually from ages 7 to 12 years in Taiwan, BMI trajectories for boys and girls were examined, with four distinct trajectories for each sex [9]. They found that the majority were in two categories–slightly underweight or healthy weight (41%) and stable healthy weight (35%), with the rest becoming obese (18%) or stable obese (7%) [9]. This study did not identify a category of children with remission of obesity. A systematic review from 2019 of weight trajectories in children identified 14 studies that investigated BMI trajectories from birth through at least age two years with risk factors of high maternal BMI and rapid early weight gain identified for excess weight, and no data explicitly describing obesity remission [10]. Many of the studies cited above used BMI z-scores in their modeling, which has been shown to be problematic when modeling trajectories for children with obesity due to the compression of a range of BMI values into a small range of BMI z-scores and the potential for changes in the transformation parameters (LMS) [11] with age and sex rather than changes in BMI [12]. As an example, Freedman et al. did an analysis showing that some 2 year old girls with severe obesity had decreases in the BMI z-score while experiencing increases in BMI or percent of the 95^th^ percentile BMI [13]. Similarly, a recent analysis examining tracking of BMI metrics in over a million children (PEDSnet) showed that BMI z-scores had the lowest intraclass correlation coefficients (ICC), and performed worst in children with obesity [14]. Distance from the median and other alternatives have been shown to have better ICCs and track growth in children with obesity better [14].

In this report, we aimed to describe the weight trajectories of school-aged children, 6–11 years of age, with a focus on those with overweight or obesity using a measure appropriate for trajectory modeling in obesity [14]. The school-age period of development is distinct from the pre-school level of influence on feeding behaviors and level of activity with marked increases in independence, and is also markedly different from adolescence and the growth acceleration associated with puberty [15]. Our first aim was to describe the weight trajectories for children with overweight and obesity, specifically focused on obesity remission or trajectories towards remission (negative slopes). Secondly, we aimed to describe the factors associated with remission or trajectories towards a healthy weight, particularly examining rurality and Latino ethnicity as this sample had substantial proportions of both overweight and obesity. Our hypothesis was that rural settings have less remission of obesity than urban settings as they have more limited access to health care (e.g. dietician) [16]. For Latino ethnicity, there are known disparities in obesity with a higher prevalence and greater degree of obesity in Latino children [17], though limited data on trajectories. Lastly, we discuss the methods and challenges of identifying groups of weight trajectories using latent class analyses or linear mixed models.

## Methods

### Data source and cleaning

We pulled data from the electronic medical record of a large hospital system in the Pacific Northwest (Portland, Oregon) using data from outpatient visits from January 1, 2015 through September 30, 2020. The initial search of the electronic medical record (EPIC) included children with data between 30 days and 18 years of age with at least two outpatient weight values recorded since January 1^st^, 2015. We then applied the Daymont et al. algorithm implemented in the R package *growthcleanr* to identify implausible values extracted from the medical record (e.g. values carried forward from a prior visit, decreases in height) [18]. Children with three or more valid observations of height and weight over at least a six-month period were included in the analysis. This project was approved by the Oregon Health & Science University Institutional Review Board, STUDY00021599. Consent was waived for this aspect of the analysis–the extraction and analysis of data from the medical record.

### Inclusion and exclusion criteria

Children with ICD-10 diagnoses of epilepsy, developmental disabilities, cerebral palsy, neoplasms including both brain and hematologic (e.g. leukemia), adrenal and other endocrine disorders, metabolic disorders, dwarfism, muscular dystrophy, spinal muscular atrophy, end stage renal disease, solid organ transplant, and receiving dialysis were excluded on the basis that these have the potential to significantly influence typical growth. To be included in the dataset, children had to be between age 6 and 11 years old at baseline with at least three valid height and weight measures over at least six months of follow-up.

### Adiposity metrics

We used several measures of adiposity in our trajectory analysis. Body mass index (BMI) was calculated in kg/m^2^ and baseline weight groups based on the Centers for Disease Control and Prevention (CDC) classification [19]. We used distance from the median BMI and percent distance from the median BMI, standardized to age, as these measures have been shown to correlate better within children over time as compared to BMI z-scores or raw BMI values, particularly in children with obesity [12]. We also used the tri-ponderal mass index (kg per meter^3^) which has also been examined as a more accurate indicator of change in adiposity [20].

### Covariates

Sex as either male or female was taken from the medical record. Race and ethnicity are self-reported with the practice being parent-report for most children in this age group. Geographic category was calculated using rural-urban community area code (RUCA) classifications with a RUCA code of 1–3 being designated urban and 4–10 being designated rural. Insurance status associated with their last visit was used to classify insurance status.

### Statistical analysis

The latent class linear mixed models (LCMM) were used to identify different trajectories of children across a range of weight in school-aged children (6 to 11 years old). For this analysis, children were followed only through age 11 for their trajectory. The latent class trajectories of percent distance from the median BMI were specified as a function of age (centered to the mean age of the cohort, 8.9 years old). Multiple LCMMs were performed using R package *lcmm (version 1*.*9*.*3)* [21] to identify the latent classes by changing the number of groups from 2 to 3 to 4, and different trajectory shapes (e.g. linear, quadratic, and cubic) with the same starting values calculated from the 1-group model. The shapes and optimal number of classes were determined by the following criteria. 1) lowest Bayesian information criteria (BIC); and 2) high mean posterior probability (>0.7). The best fitting model based on the above criteria was quadratic trajectories of three distinct classes. We used similar methods for modeling trajectories for other measures of adiposity for comparison (e.g. BMI, BMI z-scores, tri-ponderal mass index).

Logistic regression models, stratified by baseline weight group, were used to examine factors associated with membership in the majority latent class identified through LCMM. Race and ethnicity, age at baseline, insurance status, and rural/urban were all examined. Given the association of socioeconomic status and weight, we separately examined children with Medicaid insurance, which families in the United States qualify for primarily by having a low-income, as a proxy for lower socioeconomic status.

We fit linear mixed effect models with random intercept and slope, for each baseline weight group, to assess the individual relationship between adiposity and age over time. We summarized the percentage of negative predicted slopes for each baseline weight group.

We also examined the proportion in each weight stratum that changed to a different weight stratum over the observation period using the last observed follow-up time point at less than 12 years of age. A multinomial regression model was fit for each baseline weight status group examining the factors associated with being in a different weight stratum at the end of the cohort, with age, race/ethnicity, rural versus urban status, and private versus public insurance included in the models. All statistical analysis were performed using R: A Language and Environment for Statistical Computing (version 4.1.1), Vienna, Austria.

## Results

Of the 11,247 subjects included in the final sample, 410 (3.6%) were classified as underweight (<5^th^ percentile), 7,429 (66.0%) were classified as healthy weight (5^th^-<85^th^ percentile), 1,614 (14.4%) were classified as overweight (85^th^-<95^th^ percentile), and 1,794 (16.0%) were classified as obese (≥95^th^ percentile), using their baseline weight at entry into the cohort. The median length of follow-up was 2.1 years (IQR 1.2 to 3.1) for all subjects. Latino subjects made up 20.9% of the sample overall, and they had the highest rates of overweight (409/2349 or 17.4%) and obesity (689/2349 or 29.3%). (Table 1) Additionally, subjects with public (Medicaid) insurance had higher obesity (1215/4486 or 27.1%) as well as those who lived in a rural area (470/2485 or 18.9%).

**Table 1:** Demographics characteristics of the final cohort, described using baseline characteristic at entry and stratified by weight, percentages shown as percent of the total sample.

	*Overall*	*<5*^*th*^ *BMI*	*5*^*th*^*-85*^*th*^ *BMI*	*85*^*th*^*-95*^*th*^ *BMI*	*≥95*^*th*^ *BMI*	*p-value*
n	11247	410	7429	1614	1794	
Sex, n (%)						< 0.001
Female	5769 (51.3)	222 (2.0)	3744 (33.3)	806 (7.2)	997 (8.9)	
Male	5478 (48.7)	188 (1.7)	3685 (32.8)	808 (7.2)	797 (7.1)	
Age in years, mean (SD)	7.84 (1.54)	7.99 (1.60)	7.73 (1.50)	7.95 (1.57)	8.16 (1.61)	< 0.001
Race/ethnicity, n (%)						< 0.001
Hispanic or Latino	2349 (20.9)	54 (0.5)	1197 (10.6)	409 (3.6)	689 (6.1)	
White, NH	6936 (61.7)	254 (2.3)	4907 (43.6)	946 (8.4)	829 (7.4)	
Black, NH	316 (2.8)	13 (0.1)	210 (1.9)	38 (0.3)	55 (0.5)	
Asian, NH	682 (6.1)	50 (0.4)	500 (4.4)	80 (0.7)	52 (0.5)	
Other, NH	740 (6.6)	29 (3.9)	468 (4.2)	114 (1.0)	129 (1.1)	
Missing	224 (2.0)	10 (4.5)	147 (1.3)	27 (0.2)	40 (0.4)	
Geographic setting, n (%)						< 0.001
Urban or suburban	8759 (77.9)	336 (3.0)	5908 (52.5)	1191 (10.6)	1324 (11.8)	
Rural	2485 (22.1)	74 (0.7)	1518 (13.5)	423 (3.8)	470 (4.2)	
Insurance status, n (%)						< 0.001
Medicaid	4486 (39.9)	127 (1.1)	2453 (21.8)	691 (6.1)	1215 (10.8)	
Private	5371 (47.8)	242 (2.2)	3957 (35.2)	646 (5.7)	526 (4.7)	
Mixed insurance	492 (4.4)	14 (0.1)	375 (3.3)	86 (0.8)	17 (0.2)	
Missing	898 (8.0)	27 (0.2)	644 (5.7)	191 (1.7)	36 (0.3)	

NH = non-Hispanic or Latino, p-values indicate significance of either chi-square or analysis of variance comparisons between the weight-based groups

**Fig 1:**
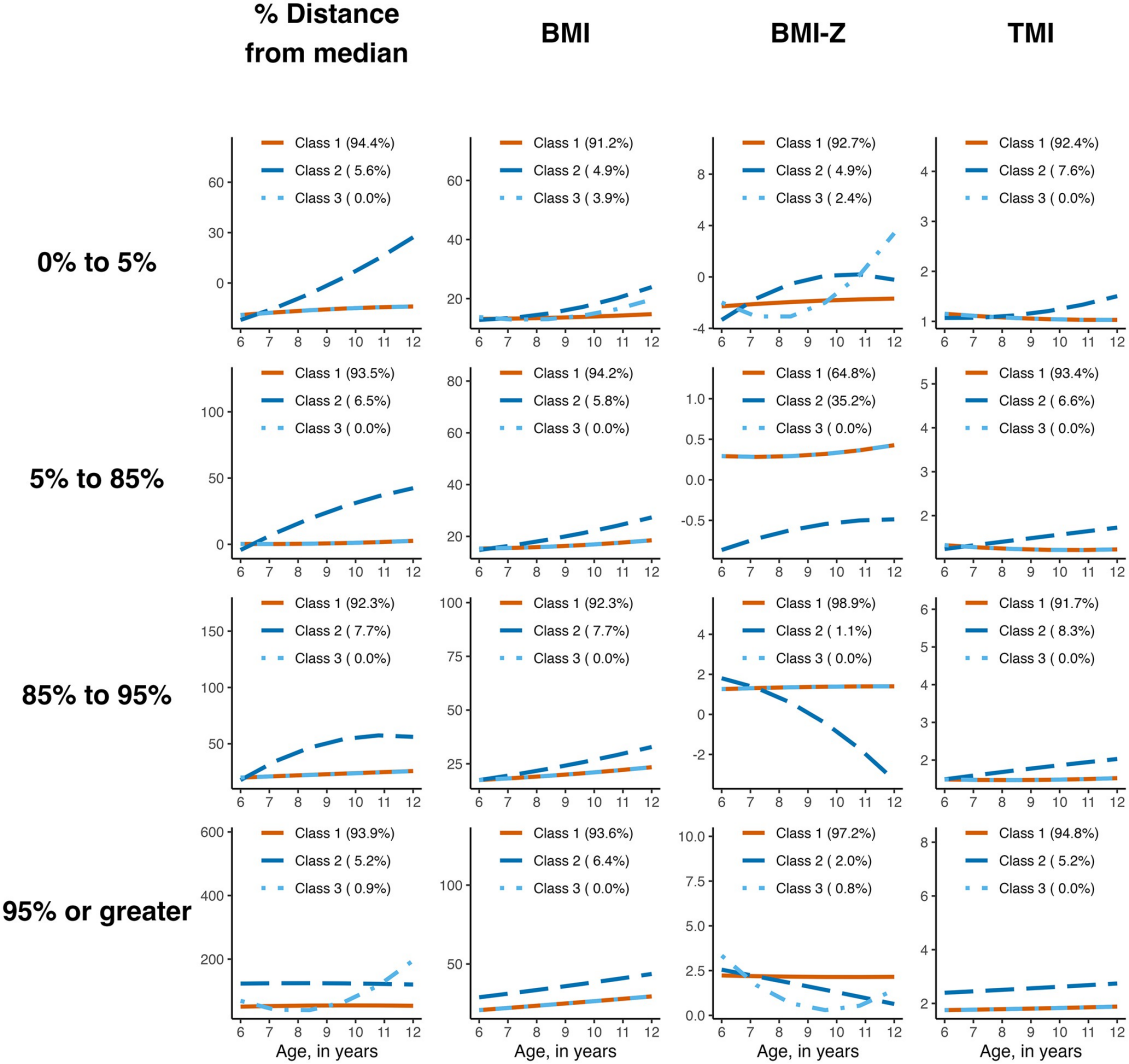
Graphical representation of the latent class linear mixed models examining different trajectories in children using various weight metrics, stratified by baseline weight status.

For each of the four baseline weight strata, we applied latent class linear mixed models using the percent distance from the median BMI to model the weight trajectories as the primary method with other metrics used for comparison (Fig 1). In children with overweight at baseline, 92.3% were in class one with a flat trajectory and 7.7% had an increasing trajectory. For children with obesity, the latent class model identified three classes with different intercepts (Fig 1), with class one (93.9%) having a flat trajectory, class two (5.2%) having a flat trajectory with a different intercept, and class three (0.9%) having a flat then upward trajectory. Findings were similar when only examining subjects with Medicaid insurance. For the <5^th^ percentile stratum, a three-class model emerged with class one having 94.4% of observations and having a flat to slightly increasing trajectory and class two (5.6%) having an upward trajectory. For children at a healthy weight, class one included 93.5% of individuals with a flat trajectory and class two with 6.5% of children having an increasing trajectory. Class three in all models had the least number of subjects and due to rounding displays as 0.0%.

We examined the predictors of being in the majority class membership—using percent distance from the median to generate trajectories. Sex was not associated with class membership. (Table 2) Race and ethnicity were only associated with trajectory class in the baseline healthy weight group with either white or non-Hispanic other categories having higher odds of being in the flat trajectory group compared with Hispanic children. Living in a rural area was not associated with latent class trajectory in any baseline weight group. Private insurance was strongly associated with membership in the flat trajectory in all groups. We also examined the Medicaid-insured group separately and found similar results for associations of race/ethnicity, rurality, sex and age. Notably, the finding of children from Hispanic families having lower odds of being in the flat trajectory group was the same within the Medicaid-only analysis. In other words, children from Hispanic families were more likely to be in the increasing trajectory group even when only examining families with public (Medicaid) insurance.

**Table 2:** Logistic regression examining odds of being in class 1 of latent class analysis, stratified by baseline weight group, estimates shown are adjusted for all of the other variables in the model.

	*<5*^*th*^ *BMI*		*5*^*th*^*-85*^*th*^ *BMI*		*85th-95*^*th*^ *BMI*		*≥95*^*th*^ *BMI*	
	OR (95% CI)	p	OR (95% CI)	p	OR (95% CI)	p	OR (95% CI)	p
Sex		0.839		0.526		0.985		0.650
Female	Reference		Reference		Reference		Reference	
Male	1.1 (0.4 to 2.7)		1.1 (0.9 to 1.3)		1.0 (0.7 to 1.5)		1.1 (0.7 to 1.6)	
Age (years)		0.005		<0.001		<0.001		<0.001
	1.6 (1.1 to 2.3)		1.6 (1.5 to 1.8)		3.3 (2.5 to 4.6)		1.3 (1.2 to 1.6)	
Race/ethnicity		0.732		0.004		0.777		0.105
Hispanic or Latino	Reference		Reference		Reference		Reference	
White, NH	0.6 (0.1 to 2.1)	0.458	1.5 (1.2 to 2.0)	0.001	1.1 (0.7 to 1.9)	0.618	1.2 (0.8 to 1.9)	0.357
Black, Asian or Other, NH	0.7 (0.1 to 3.3)	0.686	1.6 (1.1 to 2.2)	0.007	1.3 (0.6 to 2.6)	0.491	0.6 (0.4 to 1.2)	0.135
Geographic category		0.205		0.538		0.293		0.195
Urban or suburban	Reference		Reference		Reference		Reference	
Rural	0.5 (0.2 to 1.5)		1.1 (0.8 to 1.5)		1.4 (0.8 to 2.7)		0.7 (0.5 to 1.2)	
Insurance		0.008		<0.001		0.032		0.004
Medicaid	Reference		Reference		Reference		Reference	
Private	3.5 (1.4 to 9.3)		2.2 (1.8 to 2.7)		1.6 (1.0 to 2.5)		2.1 (1.2 to 3.6)	

NH = non-Hispanic or Latino; BMI = body mass index

We also present models for other commonly used measures of adiposity (Fig 1). As visualized in the figure, latent class analysis trajectories for TMI, BMI and BMI z-score differed from the trajectories for percent distance from the median. Of particular relevance is the trajectory differences for those children with overweight or obesity as the trajectory analysis for BMI z-scores identified decreasing classes for BMI z-scores that are not present in the percent distance from the median (or any other metric). The BMI and TMI trajectories were largely similar to each other with the main difference being that each of the BMI classes for overweight or obesity had more of an upward trajectory compared with the more flat trajectory of the TMI classes.

From the linear mixed model analysis, we obtained models for each baseline weight stratum with a slope for each individual estimated using the distance from the median BMI parameters. We considered slopes between -1 and 1 to be flat for the sake of categorizing subjects, which we checked by examining clinical growth curves in the medical record. Of those with overweight, 492 /1,706 (29%) had a negative slope, and 636/1999 (32%) of children with obesity had a negative slope.

**Fig 2:**
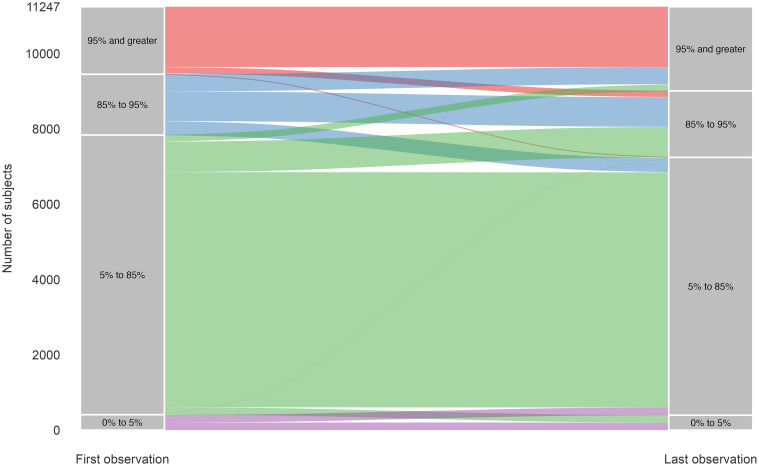
Alluvial plot showing the entry weight status of children and their weight status at exit from the cohort.

For the categorical outcomes analysis, for those starting at an obese weight in this cohort, 1,601 of 1,794 (89%) stayed in that category with only 25 (1%) moving to a healthy weight and 168 (9%) moving to overweight. (Fig 2) For those initially in the overweight category, 373 (23%) moved into a healthy weight, 786 (49%) stayed at overweight, and 455 (28%) moved to an obese weight. For those starting at a healthy weight, 6,245 of 7,429 (84%) stayed in that category with only 180 (2%) moving into the category of obesity.

In the multinomial regression model (S1 Table), children with obesity at baseline who had overweight at the end of the cohort were more likely to be white (OR 1.9, 95% CI: 1.3–2.8) or non-Hispanic, other race (OR 2.0, 95% CI: 1.2–3.4) compared with Hispanic children and to have private insurance (OR 1.7, 95% CI: 1.2–2.4) compared with public insurance. Race/ethnicity was significant in the unadjusted model for being healthy weight at the end of the cohort but no factor was significantly associated with healthy weight in the multivariable model (limited n of 25). For those with overweight at baseline, in the multivariable model, only private insurance (OR 1.4, 95% CI: 1.1–1.9) was associated with healthy weight status at the end of the cohort. Of the 28% who went from overweight to an obese weight, race/ethnicity was the only significant risk factor in the final model with both white children (OR 0.7, 95% CI: 0.5–1.0) and non-Hispanic, other race children (OR 0.6, 95% CI: 0.4–1.0) having lower odds compared with Hispanic children.

## Discussion

In this study, we found that a small proportion (1%) of children with obesity experienced remission, i.e. moved to a healthy weight, compared with 12–20% in other similar large cohort studies examining remission of obesity specifically [1, 3]. However, 24% of this sample moved from having either overweight or obesity to a healthy weight, in comparison to 8–28% in two cohorts examining those categories combined [2, 22]. The current study used a clinical health record to extract the data and generate the cohort. Therefore, this sample may have more children with clinical health problems compared to a cohort of children from a school program, from which all of the other cohort studies were derived [1–3, 22]. The mean baseline age of children in this cohort was older than most studies that have examined obesity remission. Increased age was a strong predictor of being in the flatter trajectory for those subjects with overweight or obesity. In other words, consistent with prior literature using BMI as a measure for adiposity, younger children are more likely to experience remission of obesity–nearly two thirds of children identified as having obesity at age 0–2 followed for up to three years had remission [23]. Obesity remission in one urban cohort was 29% overall, and the authors noted this was much higher among younger children; they also found no differences by race or ethnicity though it was predominantly a Black and Latino population [24]. Additionally, this study had a median follow-up of just over two years. With 32% of our sample of children with obesity having a negative slope, it is possible that a larger proportion would achieve remission if followed for a longer period of time– 9% did transition to overweight in the follow-up time period.

This study is one of the first to model growth of children with obesity using distance from the median [12]. The rationale for not using the traditional metrics of BMI z-score include its poor tracking (ICC) in children with obesity [12, 14]. This difference in methodology may also account for the differences seen in the trajectory analysis with the vast majority in each baseline weight group (95% of subjects) having a stable trajectory. A systematic review examining growth trajectory modeling in childhood found that 2 of the 14 studies that fit their inclusion criteria used a minimum of 5% of subjects in each class [10]. Across those 14 studies, they identified between three and seven growth trajectories. Notably, 7 of the 14 used BMI z-scores or BMI percentiles in their modeling, in contrast to the metric of percent distance from the median BMI used here.

Similar to the study by Luan et al. [1], private insurance as a proxy for socioeconomic status was also associated with membership in the flat category for those with obesity. While this is consistent with prior literature [25], there are notable exceptions to this finding with higher SES predicting greater adiposity for certain sub-groups, including Black children in multiple studies [26, 27].

To examine the impact of rural and urban settings on class membership, all latent class models were also adjusted for rurality. No statistically significant relationship was detected, and by our model selection criteria the models without rurality were favored. A Chinese study in 6- to 11- year-old children showed that living in a rural setting was associated with a trajectory from a healthy weight to obesity, though they did not adjust for SES [3], and a study in Canada that accounted for both rurality and SES found an association between rurality and stable obese trajectory [28].

Understanding obesity remission is important for both setting expectations in treatment and in improving our understanding of the natural history in childhood. Several studies and the US Preventive Services Task Force on Obesity have benchmarked a decrease in BMI z-score of 0.2 units as a clinically meaningful reduction in adiposity that corresponds to other clinical improvements [29–31]. The data guiding that benchmark are largely from intervention studies and use BMI z-score changes, which do not track well with changes in body adiposity for children with obesity [12, 14]. Analyses examining existing data using BMI metrics that better correspond with changes in body adiposity would be an attainable and appropriate area for further research. For example, a re-evaluation of studies previously examining BMI z-scores as the primary outcome using updated BMI metrics would be feasible and could potentially change the results and interpretation of those results. The most recent guidelines on addressing obesity from the American Academy of Pediatrics continue to suggest using BMI as a screening measure for adiposity while acknowledging its limitations and promoting a wholistic approach to health [32].

The risk factors that increase children’s risk of developing obesity have been described, both at structural and social levels as well as the family and individual behavioral levels [33–36]. The mutable family and individual-level behavioral factors associated with developing obesity include increased sedentary time, a high-calorie diet and less sleep. The association of the inverse of these risk factors with obesity remission is not as well studied and largely assumed to be true (e.g. that decreased sedentary time will lead to obesity remission). A study in the United States looking at remission of obesity between 5^th^ and 8^th^ grade showed a similarly low rate of remission to a healthy weight (1.2%) as compared with our findings; remission to either overweight or obesity was associated with measures of social-emotional functioning rather than child routines such as screen time, sleep or family dinners [37]. Clinical trials data show that changing behaviors related to diet and physical activity can have effects on weight, though most trials show some improvement and then rebound [38]. There are also seemingly contradicting data on risk factors—low birth weight has been identified as a risk factor for developing obesity [39], and yet moderate birthweight (compared to low) has been associated with obesity remission [1]. An emerging area of research in obesity relates to weight discrimination which probably contributes to continued obesity for some children who may avoid care and have adverse psychologic impacts as well [40].

Similar to prior research on weight trajectories [25], the differences observed by race and ethnicity in this sample were attenuated after accounting for socioeconomic status. This argues that the observation of an association with a worse weight trajectory, particularly for the Latino and Hispanic subgroup, may be driven by structural and economic factors, including structural racism, which has been shown previously to be associated with obesity [41]. On the other hand, children from Hispanic families were more likely to be in the increasing trajectory group when only examining those with public (Medicaid) insurance, which argues that there are other factors involved–both structural and potentially biologic. Recent evidence of genetic loci among Latino populations describes an association with an increased risk of central adiposity [42].

Prior to analysis, we expected that the LCMM models would identify 3 classes: positive, flat, and negative trajectories. The results of the analysis did not identify a negative trajectory group as a distinct class. This seemed to contradict our findings with linear mixed modelling which did show negative slopes for a percentage of subjects. This is reconciled due to a quadratic term’s inclusion in the best fitting LCMM models which recognizes the non-linear trend. The slopes from the linear mixed models represent the average trend across all ages for the subjects which does not account for non-linear trend like the LCMM models did.

Many of the prior studies that have examined weight trajectories in children have used either BMI or BMI z-score. This may be problematic as applied to trajectory analysis for those with overweight and obesity as observed by Freedman et al. [12, 43] and as shown in the analysis described here. Freedman et al. identified that children with obesity can have decreases in their BMI z-scores while increasing in adiposity–the opposite of what one would desire for a measure of adiposity. As can be seen in Fig 1, the use of BMI z-scores in particular for those with overweight or obesity identifies children as having a decreasing trajectory that was not identified by any of the other metrics. While a revisiting of those trajectory analyses may not be feasible in all cases, we posit that caution should be used in interpreting results from trajectory analyses using those metrics.

## Conclusion

We found low rates of obesity remission overall with race and SES having an association with weight trajectory and outcomes, using a school-aged cohort derived from medical record data. Further weight trajectory modeling work using distance from the median with longer periods of follow-up and incorporating additional clinical indicators in addition to BMI to characterize metabolic health are warranted using these types of data.

## Supporting information

S1 Table Multinomial regression models for last weight category, individual models by weight stratum and using the weight category at entry into the cohort as the reference, results shown as odds ratios, both unadjusted and adjusted models. (DOCX)

S1 Data (CSV)
