# Supplementary material for: Weight trajectories and obesity remission among school-aged children
Source: PLoS One. 2023 Sep 20;18(9):e0290565. doi: 10.1371/journal.pone.0290565 (PMC10511102; doi:10.1371/journal.pone.0290565)
Supplement: S1 Table — (DOCX) [file pone.0290565.s001.docx]

| **Supplemental Table**: Multinomial regression models for last weight category, individual models by weight stratum and using the weight category at entry into the cohort as the reference, results shown as odds ratios, both unadjusted and adjusted models | | | | | | | | | | |
| --- | --- | --- | --- | --- | --- | --- | --- | --- | --- | --- |
|  |  |  | *Baseline <5^th^ BMI* | | | | *Baseline 5^th^-85^th^ BMI* | | | |
| Final weight category |  |  | Unadjusted OR (95% CI) | p | Adjusted OR (95% CI) | p | Unadjusted OR (95% CI) | p | Adjusted OR (95% CI) | p |
| *<5^th^ BMI* | Sex | Female |  |  |  |  | Reference level |  | Reference level |  |
|  |  | Male |  |  |  |  | 0.8 (0.6 to 1.0) | 0.089 | 0.8 (0.6 to 1.1) | 0.131 |
|  | Age |  |  |  |  |  | 1.0 (0.9 to 1.1) | 0.454 | 1.0 (0.9 to 1.1) | 0.569 |
|  | Race | Hispanic or Latino |  |  |  |  | Reference level |  | Reference level |  |
|  |  | NH, White |  |  |  |  | 1.4 (0.9 to 2.3) | 0.119 | 1.3 (0.8 to 2.2) | 0.252 |
|  |  | NH, Black or Asian or Other |  |  |  |  | 1.7 (1.0 to 2.8) | 0.071 | 1.5 (0.9 to 2.7) | 0.155 |
|  | Rural/urban | Urban/suburban |  |  |  |  | Reference level |  | Reference level |  |
|  |  | Rural |  |  |  |  | 0.8 (0.5 to 1.1) | 0.208 | 1.0 (0.7 to 1.6) | 0.861 |
|  | Insurance | Medicaid |  |  |  |  | Reference level |  | Reference level |  |
|  |  | Private |  |  |  |  | 1.4 (1.0 to 1.9) | 0.063 | 1.3 (0.9 to 1.8) | 0.173 |
| *5^th^-85^th^ BMI* | Sex | Female | Reference level |  | Reference level |  |  |  |  |  |
|  |  | Male | 0.9 (0.6 to 1.4) | 0.777 | 1.0 (0.7 to 1.5) | 0.921 |  |  |  |  |
|  | Age |  | 0.9 (0.8 to 1.1) | 0.229 | 1.0 (0.8 to 1.1) | 0.497 |  |  |  |  |
|  | Race | Hispanic or Latino | Reference level |  | Reference level |  |  |  |  |  |
|  |  | NH, White | 0.7 (0.4 to 1.2) | 0.199 | 0.6 (0.3 to 1.1) | 0.113 |  |  |  |  |
|  |  | NH, Black or Asian or Other | 0.4 (0.2 to 0.7) | 0.005 | 0.3 (0.2 to 0.7) | 0.005 |  |  |  |  |
|  | Rural/urban | Urban/suburban | Reference level |  | Reference level |  |  |  |  |  |
|  |  | Rural | 1.1 (0.7 to 1.8) | 0.673 | 1.0 (0.6 to 1.8) | 0.999 |  |  |  |  |
|  | Insurance | Medicaid | Reference level |  | Reference level |  |  |  |  |  |
|  |  | Private | 0.8 (0.5 to 1.3) | 0.401 | 1.0 (0.6 to 1.6) | 0.97 |  |  |  |  |
| *85^th^-95^th^ BMI* | Sex | Female | Reference level |  | Reference level |  | Reference level |  | Reference level |  |
|  |  | Male | 0.4 (0.0 to 3.7) | 0.405 | 0.5 (0.0 to 4.9) | 0.532 | 0.8 (0.7 to 1.0) | 0.019 | 0.8 (0.7 to 1.0) | 0.014 |
|  | Age |  | 1.4 (0.6 to 3.0) | 0.456 | 1.4 (0.6 to 3.3) | 0.48 | 1.0 (1.0 to 1.1) | 0.075 | 1.0 (1.0 to 1.1) | 0.173 |
|  | Race | Hispanic or Latino | Reference level |  | Reference level |  | Reference level |  | Reference level |  |
|  |  | NH, White | 0.2 (0.0 to 2.8) | 0.212 | 0.3 (0.0 to 6.4) | 0.474 | 0.5 (0.4 to 0.6) | < 0.001 | 0.6 (0.5 to 0.7) | < 0.001 |
|  |  | NH, Black or Asian or Other | 0.7 (0.1 to 8.3) | 0.788 | 1.2 (0.1 to 16.3) | 0.871 | 0.6 (0.4 to 0.7) | < 0.001 | 0.7 (0.5 to 0.9) | 0.01 |
|  | Rural/urban | Urban/suburban | Reference level |  | Reference level |  | Reference level |  | Reference level |  |
|  |  | Rural | 0.0 (0.0 to Inf) | 0.775 | 0.0 (0.0 to 0.0) | < 0.001 | 1.3 (1.1 to 1.5) | 0.005 | 1.0 (0.8 to 1.3) | 0.726 |
|  | Insurance | Medicaid | Reference level |  | Reference level |  | Reference level |  | Reference level |  |
|  |  | Private | 0.1 (0.0 to 1.4) | 0.101 | 0.1 (0.0 to 1.6) | 0.118 | 0.5 (0.5 to 0.6) | < 0.001 | 0.6 (0.5 to 0.7) | < 0.001 |
| *≥95^th^ BMI* | Sex | Female |  |  |  |  | Reference level |  | Reference level |  |
|  |  | Male |  |  |  |  | 0.6 (0.4 to 0.8) | < 0.001 | 0.5 (0.4 to 0.7) | < 0.001 |
|  | Age |  |  |  |  |  | 1.0 (0.9 to 1.1) | 0.813 | 1.0 (0.9 to 1.1) | 0.796 |
|  | Race | Hispanic or Latino |  |  |  |  | Reference level |  | Reference level |  |
|  |  | NH, White |  |  |  |  | 0.7 (0.5 to 1.0) | 0.053 | 0.9 (0.6 to 1.4) | 0.608 |
|  |  | NH, Black or Asian or Other |  |  |  |  | 0.6 (0.3 to 1.0) | 0.045 | 0.8 (0.4 to 1.4) | 0.381 |
|  | Rural/urban | Urban/suburban |  |  |  |  | Reference level |  | Reference level |  |
|  |  | Rural |  |  |  |  | 1.3 (0.9 to 1.8) | 0.197 | 0.9 (0.6 to 1.4) | 0.698 |
|  | Insurance | Medicaid |  |  |  |  | Reference level |  | Reference level |  |
|  |  | Private |  |  |  |  | 0.4 (0.3 to 0.5) | < 0.001 | 0.4 (0.3 to 0.5) | < 0.001 |

|  |  |  | *Baseline 85^th^-95^th^ BMI* | | | | *Baseline ≥95^th^ BMI* | | | |
| --- | --- | --- | --- | --- | --- | --- | --- | --- | --- | --- |
| Final weight category |  |  | Unadjusted OR (95% CI) | p | Adjusted OR (95% CI) | p | Unadjusted OR (95% CI) | p | Adjusted OR (95% CI) | p |
| *5^th^-85^th^ BMI* | Sex | Female | Reference level |  | Reference level |  | Reference level |  | Reference level |  |
|  |  | Male | 1.0 (0.8 to 1.3) | 0.803 | 1.0 (0.8 to 1.3) | 0.881 | 0.7 (0.3 to 1.6) | 0.427 | 0.7 (0.3 to 1.6) | 0.425 |
|  | Age |  | 1.0 (0.9 to 1.1) | 0.54 | 1.0 (0.9 to 1.1) | 0.468 | 0.8 (0.7 to 1.1) | 0.21 | 0.9 (0.7 to 1.1) | 0.229 |
|  | Race | Hispanic or Latino | Reference level |  | Reference level |  | Reference level |  | Reference level |  |
|  |  | NH, White | 1.5 (1.1 to 2.1) | 0.01 | 1.1 (0.8 to 1.6) | 0.458 | 2.9 (1.0 to 7.9) | 0.04 | 2.6 (0.9 to 7.4) | 0.069 |
|  |  | NH, Black or Asian or Other | 1.1 (0.7 to 1.7) | 0.618 | 0.8 (0.5 to 1.3) | 0.337 | 2.5 (0.7 to 9.5) | 0.17 | 2.6 (0.7 to 10.1) | 0.167 |
|  | Rural/urban | Urban/suburban | Reference level |  | Reference level |  | Reference level |  | Reference level |  |
|  |  | Rural | 0.9 (0.7 to 1.2) | 0.4 | 1.0 (0.7 to 1.4) | 0.929 | 1.1 (0.4 to 2.6) | 0.887 | 1.1 (0.5 to 2.8) | 0.778 |
|  | Insurance | Medicaid | Reference level |  | Reference level |  | Reference level |  | Reference level |  |
|  |  | Private | 1.4 (1.1 to 1.8) | 0.011 | 1.4 (1.1 to 1.9) | 0.02 | 1.6 (0.7 to 3.7) | 0.234 | 1.3 (0.6 to 3.0) | 0.546 |
| *85^th^-95^th^ BMI* | Sex | Female |  |  |  |  | Reference level |  | Reference level |  |
|  |  | Male |  |  |  |  | 1.3 (0.9 to 1.8) | 0.136 | 1.3 (0.9 to 1.8) | 0.119 |
|  | Age |  |  |  |  |  | 1.1 (0.9 to 1.2) | 0.319 | 1.1 (1.0 to 1.2) | 0.256 |
|  | Race | Hispanic or Latino |  |  |  |  | Reference level |  | Reference level |  |
|  |  | NH, White |  |  |  |  | 2.2 (1.5 to 3.2) | < 0.001 | 1.9 (1.3 to 2.8) | 0.002 |
|  |  | NH, Black or Asian or Other |  |  |  |  | 2.3 (1.4 to 3.8) | 0.002 | 2.0 (1.2 to 3.4) | 0.011 |
|  | Rural/urban | Urban/suburban |  |  |  |  | Reference level |  | Reference level |  |
|  |  | Rural |  |  |  |  | 0.7 (0.5 to 1.1) | 0.099 | 0.8 (0.5 to 1.2) | 0.258 |
|  | Insurance | Medicaid |  |  |  |  | Reference level |  | Reference level |  |
|  |  | Private |  |  |  |  | 2.2 (1.6 to 3.0) | < 0.001 | 1.7 (1.2 to 2.4) | 0.003 |
| *≥95^th^ BMI* | Sex | Female | Reference level |  | Reference level |  |  |  |  |  |
|  |  | Male | 1.0 (0.8 to 1.3) | 0.752 | 0.9 (0.7 to 1.2) | 0.686 |  |  |  |  |
|  | Age |  | 1.0 (0.9 to 1.1) | 0.602 | 1.0 (0.9 to 1.0) | 0.334 |  |  |  |  |
|  | Race | Hispanic or Latino | Reference level |  | Reference level |  |  |  |  |  |
|  |  | NH, White | 0.7 (0.5 to 0.9) | 0.009 | 0.7 (0.5 to 1.0) | 0.023 |  |  |  |  |
|  |  | NH, Black or Asian or Other | 0.6 (0.4 to 0.9) | 0.015 | 0.6 (0.4 to 1.0) | 0.043 |  |  |  |  |
|  | Rural/urban | Urban/suburban | Reference level |  | Reference level |  |  |  |  |  |
|  |  | Rural | 1.0 (0.8 to 1.3) | 0.971 | 0.9 (0.6 to 1.2) | 0.455 |  |  |  |  |
|  | Insurance | Medicaid | Reference level |  | Reference level |  |  |  |  |  |
|  |  | Private | 0.8 (0.7 to 1.1) | 0.174 | 0.9 (0.7 to 1.2) | 0.609 |  |  |  |  |

NH = Non-Hispanic, non-Latino; BMI = body mass index
